# Experiences and perceptions of IBD-related psychological distress in persons living with IBD

**DOI:** 10.1093/jcag/gwag009

**Published:** 2026-03-07

**Authors:** Natalie Willett, Courtney Heisler, Noelle Rohatinsky, Sophie Farina, Michael Stewart, Tiffany Shepherd, Barbara Currie, Kelly Phalen, Jessica Robar, Thea Huard, Emily Neil, Jennifer L Jones

**Affiliations:** Nova Scotia Health, Division of Digestive Care and Endoscopy, Halifax, B3H 2Y9, Canada; Nova Scotia Health, Division of Digestive Care and Endoscopy, Halifax, B3H 2Y9, Canada; University of Saskatchewan, Saskatoon, S7N 5A2, Canada; Nova Scotia Health, Division of Digestive Care and Endoscopy, Halifax, B3H 2Y9, Canada; Nova Scotia Health, Division of Digestive Care and Endoscopy, Halifax, B3H 2Y9, Canada; Dalhousie University, Department of Medicine, Halifax, B3H 4R2, Canada; Nova Scotia Health, Division of Digestive Care and Endoscopy, Halifax, B3H 2Y9, Canada; Nova Scotia Health, Division of Digestive Care and Endoscopy, Halifax, B3H 2Y9, Canada; Nova Scotia Health, Division of Digestive Care and Endoscopy, Halifax, B3H 2Y9, Canada; Nova Scotia Health, Division of Digestive Care and Endoscopy, Halifax, B3H 2Y9, Canada; Patient Research Partner at Nova Scotia Health, Halifax, B3H 2Y9, Canada; Patient Research Partner at Nova Scotia Health, Halifax, B3H 2Y9, Canada; Nova Scotia Health, Division of Digestive Care and Endoscopy, Halifax, B3H 2Y9, Canada; Dalhousie University, Department of Medicine, Halifax, B3H 4R2, Canada

**Keywords:** inflammatory bowel disease, qualitative, interviews, patient oriented

## Abstract

**Background:**

IBD-related psychological distress (IBD-PD) refers to the emotional impact of IBD and is associated with increased IBD activity. The inability to provide high-quality, person-centred care for IBD-PD that is proportional to clinical need is a significant healthcare gap in the Canadian healthcare system. The aim of this study was to generate stakeholder-derived data about patient experiences with IBD-PD in Nova Scotia, Canada.

**Methods:**

Virtual semi-structured interviews took place between October 2021 and March 2022. The interview script was developed iteratively with researchers, IBD care providers, and patient research partners. Questions were designed to assess perceptions and experiences with IBD-PD. Adult IBD patients were recruited from IBD clinics. Using thematic analysis, codes were generated to identify themes.

**Results:**

Nineteen individuals were approached to participate. The total number of participants recruited and enrolled was 14. The mean participant age was 37.6 years (range 23-57) with 57.1% as female (8/14). The following themes emerged: (1) There are specific triggers for IBD-PD such as hospital settings, social isolation, and stress (2) Times in a patients journey that are psychologically distressing include: initial diagnosis, transitions to new medication or new treatment, and surgery (3) some participants achieved psychological wellbeing while living with IBD and reported their experience gave them new perspective on life and more empathy and compassion.

**Conclusions:**

There are many triggers for IBD-PD and timepoints associated with IBD-PD throughout a patient’s disease course. It is important to understand IBD-PD from the patient perspective, so that future interventions meet patient-specific needs.

## Introduction

The rapid growth in inflammatory bowel disease (IBD) prevalence and management complexity continues to expand year over year.^1^^,^^2^ In this decade, it is projected that an estimated 470,000 Canadians will be diagnosed with IBD.^1^ Canada has the highest observed prevalence rates of IBD in the world.^3^

IBD-related psychological distress (IBD-PD) refers to the emotional reactions to a chronic disease like IBD, which can be associated with increased stress, comorbid mental health disorders, and symptoms of active disease.^4^^,^^5^ Mental health conditions in IBD are also associated with increased primary care and ER visits.^6^ The COVID-19 pandemic brought awareness to the growing mental health crisis in the general population and in those with IBD.^7^^,^^8^ Among the IBD population alone, many IBD patients experience some form of IBD-PD at some point during their IBD journey, and patients feel there is inadequate access to mental health supports for this distress.^9^^,^^10^ Patients feel their mental and emotional health needs are not currently being met in their routine IBD care.^11–13^ Routine screening and referral for mental health problems are clinical needs that are not being met and more implementation studies on routine mental health assessments in IBD clinics are needed.^14^ Despite this care gap, IBD patients indicate high levels of comfort with discussing psychological distress with their IBD care team compared to receiving support from formalized mental health professionals.^15^^,^^16^ In the United States, only one-third of patients with IBD-PD are able to access mental health care. In fact, a predictive factor of psychological distress is being ‘not able to find a healthcare provider’.^5^

A recent meta-analysis suggests there is a high prevalence of anxiety and depressive symptoms in IBD patients, and the prevalence of these symptoms (anxiety and depression) is higher in those with active IBD.^17^ From a gut-brain behaviour outcomes framework, psychological distress contributes to IBD disease activity over time and exacerbates symptoms.^18^ Psychological distress in IBD has been linked to stressful life events and lack of social support, with social support having a protective effect on the degree of distress.^19^ IBD patients have higher levels of anxiety, stress, and depression compared to non-IBD controls and levels of distress correlate with proinflammatory gene expression in ileal tissue.^20^

Psychological therapies for IBD-PD are broadly classified into psychotherapies, relaxation techniques and education, which are often used in combination to improve quality of life and emotional state.^21^ Overall, psychotherapy may improve quality of life and symptoms and anxiety and depression, Interventions that use patient education and relaxation may also have small positive effects on QoL, but more well-powered randomized controlled trials on inflammation and bodily effects of psychological interventions in IBD are needed.^21^

Due to a disproportionate population need for mental health services compared to the number of mental health professionals available, many seeking care are faced with daunting wait lists and a lack of clarity about when they will be able to access a mental health professional.^10^^,^^22^ Access to mental health support is needed because living with the disease can be stigmatizing, stressful, and isolating.^23^ With systemic healthcare barriers to accessing formalized supports for psychological distress, patients are often left to self-manage or rely on informal supports.^24^

Ensuring the provision of high-quality, patient-centred care for IBD-PD that is proportionate to the level of distress severity will address key evidence-practice gaps in our healthcare system. The Nova Scotia Collaborative IBD program (NSCIBD) has built a research program called *IBD Strong* that will design, implement, and evaluate multifaceted, stepped intensity cognitive behavioral therapy-based interventions for IBD-PD. The IBD Strong research network is a multidisciplinary network consisting of patients, researchers, psychologists, implementation scientists, allied health professionals, and clinicians. This project’s aim is to generate stakeholder-derived data about patient experiences with IBD-PD, and these patient experiences will inform the development and implementation of future evidence-based, patient-centred interventions for IBD-PD.

## Methodology

### Participants and interviews

Adults diagnosed with IBD were approached and recruited from IBD clinics at the QEII Health Sciences Centre in Halifax, Nova Scotia. Interview scripts were iteratively developed with collaboration from physicians, nurses, patients, and researchers. Across all interviews conducted between 2021 and 2022, participants with varying degrees of IBD disease severity were asked about their experiences, the impact, and the causes of psychological distress related to their IBD. Interviews were co-facilitated by a research associate with experience facilitating national focus groups (CH, female) and a patient research partner (TH, female), each 1 hour in length. No repeat interviews were carried out. No relationship between the participants and facilitators was established prior to study commencement, and the research associate explained the goals of this research during the consent process. All audio from each interview was recorded on the NSH Zoom for Healthcare platform; no field notes were taken at the time of recording. Audio was transcribed using software and cleaned by research assistants. Transcripts were not sent or shared with participants and they did not give feedback on thematic findings post-analysis.

### Analysis

Interview recordings were transferred via Province of Nova Scotia Secure File Transfer to the Canadian Hub for Applied and Social Research (CHASR) at the University of Saskatchewan. All identifying information was removed. The initial themes and coding were developed while reviewing hard copies of the first 4 transcripts. Transcripts were then uploaded into NVivo Pro (Version 12) and each interview was coded by topic to further refine the initial coding following the 6-step thematic analysis process outlined by Braun and Clarke 2006.^25^ Codes for participant responses from the 14 interviews were tracked. While some coding tracked specific questions, most coding was done across participant responses and settings. The Consolidated Criteria for Reporting Qualitative Research (COREQ checklist for this project is available online (supplementary material).

## Results

Nineteen adults with IBD were approached to take part in this study and consent forms were sent, and 14 individuals were ­interviewed in this study. Five of the 19 were lost to follow-up and did not complete the interview as scheduled. The mean ­participant age was 37.6 years (range 23-57). The majority of participants were female (57.1%, or 8/14), and most participants worked full-time (64.3%, or 9/14). All had completed at least a high school education. Diagnosis of Crohn’s disease comprised 28.6% (4/14) of the sample, Ulcerative colitis also comprised 28.6% (4/14) of the sample, and the remainder of participants had IBD undifferentiated. The remaining proportion of the sample was made up of individuals who had IBD unspecified. No participants in the group identified as a member of a minority. Twenty-eight percent lived in a rural area (4/14), and 71.4% (10/14) lived in an urban area.

### Theme 1: Triggers and causes of psychological distress

Participants identified the onset of symptoms, their initial diagnosis, healthcare environments, and challenges related to finding medication as triggers of psychological distress. Participants discussed triggers that lead to IBD flares and complications from IBD as well.

#### Stress

Large-scale life events and significant changes or moves were identified as contributing factors to psychological stress and a trigger for IBD flares. Participants referenced travel, family-related stressors, and starting a new school as triggering events.

This past summer my older sister was getting married, and she is in New York and there was a lot of stress around would I be able to come….I’m getting married on Monday and one of my really good friends is getting married in a couple of weeks so automatically this spring I was recognizing those were three big things, plus my grandmother’s unwell so there were a lot of things going on. I recognize those stressors; I recognize that even just one of those could be enough to send me into a flare. (P01)

#### Healthcare settings

Participants identified healthcare settings as a trigger for anxiety and stress that could cause a flare. Past negative experiences with healthcare and trauma related to hospitals and treatment were identified as contributing factors**.** Healthcare settings were associated with discomfort, fear, and were labelled by one patient as cold and unfeeling.

I feel like, just because my impression of the healthcare system is that it’s cold and unfeeling. The broader system, not the people in it necessarily, but just the broader system. I probably wouldn’t bring it up because everything so stressed already that I would just be like okay like there’s nothing for me even if I brought it up …And I’m going to appointments which suck, and I hate them. They create the emotional distress (P05)

#### Social situations

Social situations at work or with friends, and large events or holidays, were identified as challenging situations that could trigger psychological distress. Fear of judgment from others for taking multiple trips to the bathroom was also a concern of participants. Participants also reflected that their disease made pursuing and maintaining intimate relationships more difficult at times.

I remember those days as being challenging to some degree, whether it was relationships or work. When you’re making multiple trips to the washroom know people look at you and think “what’s wrong with that guy? (P10)

#### Symptoms

For many participants, the symptoms of IBD were a cause of psychological distress, particularly prior to their diagnosis. Participants often reported blood in stool as being a symptom that caused distress.

### Theme 2: Diagnosis, medications, and treatment

In addition to the physical stressors, the disease itself, the diagnostic and treatment process can take time, which is an additional factor that causes distress. There were often specific examples and situations from the time when they received their diagnosis that they remembered as traumatic.

At the time when I had just been diagnosed with IBD. Dr. [name removed] specifically I remember, after the first time I came back after I’d gotten my colonoscopy and she sat us down to tell me that I had, at the time they thought it was ulcerative colitis, but just recently I was re-diagnosed with Crohn’s… she sat me down the first time and told me I had ulcerative colitis, and right after she told me that, I was kind of like “oh my god. (P07)

For one participant living in a small community, talking about IBD with the local doctor and the fear of others in the community finding out was a concern prior to diagnosis.

I ended up going to the neighboring communities’ doctor because I didn’t even want to talk about this with my own doctor. Um, whereas I think people know more about the disease now, they’re more… open about how they feel how the disease impacts them. I can’t say that I’ve experienced anything traumatic since then, but I think it was the anxiety associated with “what do I have?”. Until I was diagnosed, I knew what my path was going to look like, I had a rough road for the first 10 years before my condition was stabilized… (P10)

Transitions to new medication or a new treatment were particularly hard for many participants, who identified that they felt distress. Barriers to receiving medication can contribute to additional stress that adds to the difficulty of navigating the diagnosis and treatment. Medication changes were identified as a challenging time psychologically, especially when combined with other life stressors.

“When I was first diagnosed I didn’t have a ton of follow up it, was just kind of like “take these drugs”… so I was taking them for kind of a long time and they start to make me feel nauseated in the morning. So I had to stop taking them and then I was good for a bit and then the bleeding came back after a little while and I was concerned about it because I was told that maybe it would be temporary and it was maybe due to my pregnancy. (P11)

### Theme 3: Psychological wellbeing in the face of IBD

When asked when they have experienced psychological wellbeing during their experience of IBD, one participant said that no aspect of IBD contributed to their psychological wellness. Some participants approached the concept of psychological wellbeing as something achieved through acceptance, while others emphasized their resilience, or their capacity for understanding and empathy. For some participants, IBD was seen as an occasional interruption in their life, being able to compartmentalize or forget about negative symptoms. Other participants describe having gained some confidence and developing new coping skills and abilities during their struggle with IBD. Two participants framed psychological wellness as the way they have been able to contribute to helping and understanding others. Participants also felt well after a significant disease intervention, such as bowel surgery: 2 participants described the period post-surgery as a positive time when they felt physically and psychologically well.

Having lived with this disease for as long as I have and being open about it has allowed me to, kind of, share my journey and my experience with other people. So, whether that means people who have similar symptoms or even a similar diagnosis and wonder about everything from medication to eating habits, those would be the main things… I’d say when you know that you can help someone out, that feels good. (P10)

Two participants were on extreme ends of the spectrum in relation to acceptance and denial. One participant repeatedly said that the moment when they accepted their diagnosis brought them great psychological wellbeing, while another felt that staying in denial helped them. Some participants built resilience along with a method of compartmentalizing and forgetting about IBD until another flare-up. Others felt that acknowledging and coming to terms with their diagnosis was their path to psychological wellbeing.

## Discussion

These interviews asked participants about their lived experiences with IBD, episodes of IBD-PD, and perceptions of mental health in the context of living with IBD. Triggers for IBD-PD identified by participants (stress, triggering hospital settings, isolation, symptoms, gatherings, and social situations) have been well-documented.^26^^,^^27^ Symptoms and disease flares as reported triggers of IBD-PD are consistent with a recent meta-analysis of reduced quality of life in people with active IBD.^28^ Qualitative work done by Mikocka-Walus et al.^29^ revealed that participants who reported a negative psychological impact from the disease attributed these feelings to the limitations their disease imposed on them in the domains of performing daily activities and engaging in social events. Self-efficacy has been associated with decreased incidence of self-reported flares, clinical symptoms, and daily life impact.^30^^,^^31^ IBD is known to affect recreational or professional activities, and relationships with relatives or friends.^32^ Participants also report having difficulties related to reintegrating into social settings with IBD, and suffering from feelings of loneliness.^33^ Furthermore, there is a positive correlation between loneliness and social isolation in people with IBD, with loneliness being associated with female gender and older age.^34^

Some participants in this study reported resilience throughout their experiences of IBD-PD and living with IBD, which is a novel and growing area of research in IBD. Resilience in chronic illness can be explained by subjective wellbeing homeostasis (SWH) theory where research can quantify a ‘resilience effect’, where the linear relationship between increasing psychological stressors and decreases in subjective wellbeing breaks, and there is a plateau.^35^ It is theorized here that individuals have a ‘homeostatically protected mood’. Lyall et al.^36^ used quantitative methods and were able to identify a resilience effect in people living with CD, aligning with the theory that individuals can maintain healthy levels of protected mood and that this mood homeostasis can buffer the impact of temporary depressive symptoms. The 2023 Impact of Inflammatory Bowel Disease in Canada report identified that resilience is associated with improved mental health outcomes, improved IBD outcomes, and points out that it is a promising target for future interventions.^37^ Participants in this study have explained that their IBD has sometimes led them to be more grateful for their own life and the days they have without symptoms, as well as brought about a desire to help others through their struggles. Post-traumatic growth has been explored in IBD, and researchers have found this type of growth to be associated with illness cognitions, such as acceptance and helplessness.^38^ While living with IBD may consequently lead to episodes of IBD-PD, Lawton^39^ comments that it is a mistake to conceptualize the IBD experience as singularly and exclusively negative, and that IBD is a multidimensional experience that varies from person to person.

Thematic analysis revealed that there were certain times throughout the course of their IBD journey where participants would have benefited from having mental health support. Sigethy et al.^16^ in their White paper, argue that there is ample evidence to suggest a combined, integrated care approach for IBD patients that incorporates psychosocial interventions in addition to standard care be made valuable to IBD patients. IBD patients who have psychological distress report being significantly less satisfied with their IBD care, and an integrated biopsychosocial model of health care is preferred by patients.^40^ IBD-PD psychological treatments that are group-based or ones that involve talking to others with IBD (such as peer mentoring programs) provide patients with a chance to connect with other IBD patients. This may improve IBD-PD by reducing a patient’s sense of isolation.

Our findings reveal that the timing of access to IBD PD care may also be important. Participants are in most need of psychological support at specific time points: during diagnosis, symptom flares, and treatment initiation. There is data to support these findings. A recent meta-analysis revealed that IBD patients face an increased risk of both anxiety and depression following initial diagnosis.^41^ Authors also state that more temporal studies quantifying the risk of anxiety and depressive symptoms at different time points throughout the disease course are needed. Following diagnosis of IBD, the incidence of depression, anxiety, insomnia, and deliberate self-harm is higher in individuals with IBD compared to healthy controls.^42^ There is also a significantly higher risk for patients to discontinue biologic medications if they have anxiety or mood disorders prior to initiating the medication.^43^ People living with mood disorders are more likely to have gastrointestinal somatic symptoms and associate them with a new therapy. Similar findings appear in the IBD pediatric population as well.^44^

Events that participants described as the most distressing throughout the course of their IBD journey (initial onset of symptoms, flares, diagnosis, referrals for surgery) are often sudden and unpredictable. In cases like these, it may be impossible to arrange timely mental health support through traditional channels like psychiatric referrals. That limitation should be considered when planning interventions and implementation strategies.

This work should be expanded to include the area of ostomies, as the needs and experiences of people living with ostomies can differ from the general IBD population in significant ways. Although ostomy surgery allows IBD patients to participate in activities, improve physical quality of life, and allows them to go out in public, there are additional challenges such as body image issues and living with a hidden disability.^45^^,^^46^ Those living with ostomies might benefit from a modified support plan, and it is important to gather data through community engagement to explore their perceptions and experiences of IBD-PD.

These findings reflect the experiences and perceptions of Nova Scotians living with IBD. All healthcare interventions should be tailored to patients needs and these needs differ based on patient subgroup and location. This underscores the importance of representative provincial patient engagement that is sensitive to local contextual factors. Access to mental health supports and experiences of people living with IBD vary by geographical location due to local municipal and provincial government laws and policies, and patient demographics.^47–50^ Underrepresented subgroups that should be engaged include indigenous peoples, racialized or immigrant communities, marginalized communities, rural communities, and people with limited access to care. Undertaking this work as early steps to designing future IBD-PD interventions will promote high-quality, person-centred care that is equitable and personalized, optimizing the likelihood of improved patient outcomes for all Canadians suffering from IBD-PD.

### Strengths and limitations

There are various strengths to this study design: the study sample spans different age groups, genders, geographic locations, and disease types. Additionally, the interview questions were designed using a multidisciplinary approach involving iterative feedback from researchers, IBD care providers, and patient research partners. This study also incorporated a patient research partner into the interview process, which participants appreciated. Qualitative data provided from these interviews also allowed us to explore triggers and experiences of IBD-PD in a more in-depth manner, compared to quantitative methods such as surveys.

This study has key limitations. The sample of interviewees with IBD lacked ethnic diversity, and researchers faced difficulty in recruitment from key marginalized groups. We lack data on reasons for loss to follow-up of enrollees, so we cannot comment on how selection bias may be operating. Future studies measuring IBD-PD quantitatively are needed to capture the degree of distress patients face and identify additional events and timepoints at which this distress is most likely to coincide.

## Conclusion

There are many important triggers (predictable and unpredictable) of IBD-PD. These triggers include stress, healthcare settings, pandemic and disease-associated social isolation, and social gatherings. Patients feel that having more support would have helped them through difficult periods where these triggers were present. A pattern of psychological distress related to medications and initial IBD diagnosis suggests the need for mental health support at the onset of IBD diagnosis, flares, and treatment change. Although living with IBD can involve episodes of IBD-PD, some participants were able to achieve psychological wellness through resilience, and the impact of IBD on patients’ mental health was not always negative. It is important to understand patients’ experiences living with IBD. Patient engagement through these interviews will influence the design and implementation of programmatic interventions designed with the patients’ direct input, which incorporates personal preferences.

## Supplementary Material

gwag009_Supplementary_Data

## Data Availability

The data that support the findings of this study are not openly available due to reasons of maintaining confidentially for participants.
